# Supplementary material for: HSP90 differentially stabilizes plant ABCB-type auxin transporters on the plasma membrane
Source: Nat Commun. 2025 Sep 30;16:8643. doi: 10.1038/s41467-025-63780-w (PMC12484997; doi:10.1038/s41467-025-63780-w)
Supplement: Supplementary file 1 — Supplementary Information [file 41467_2025_63780_MOESM1_ESM.pdf]

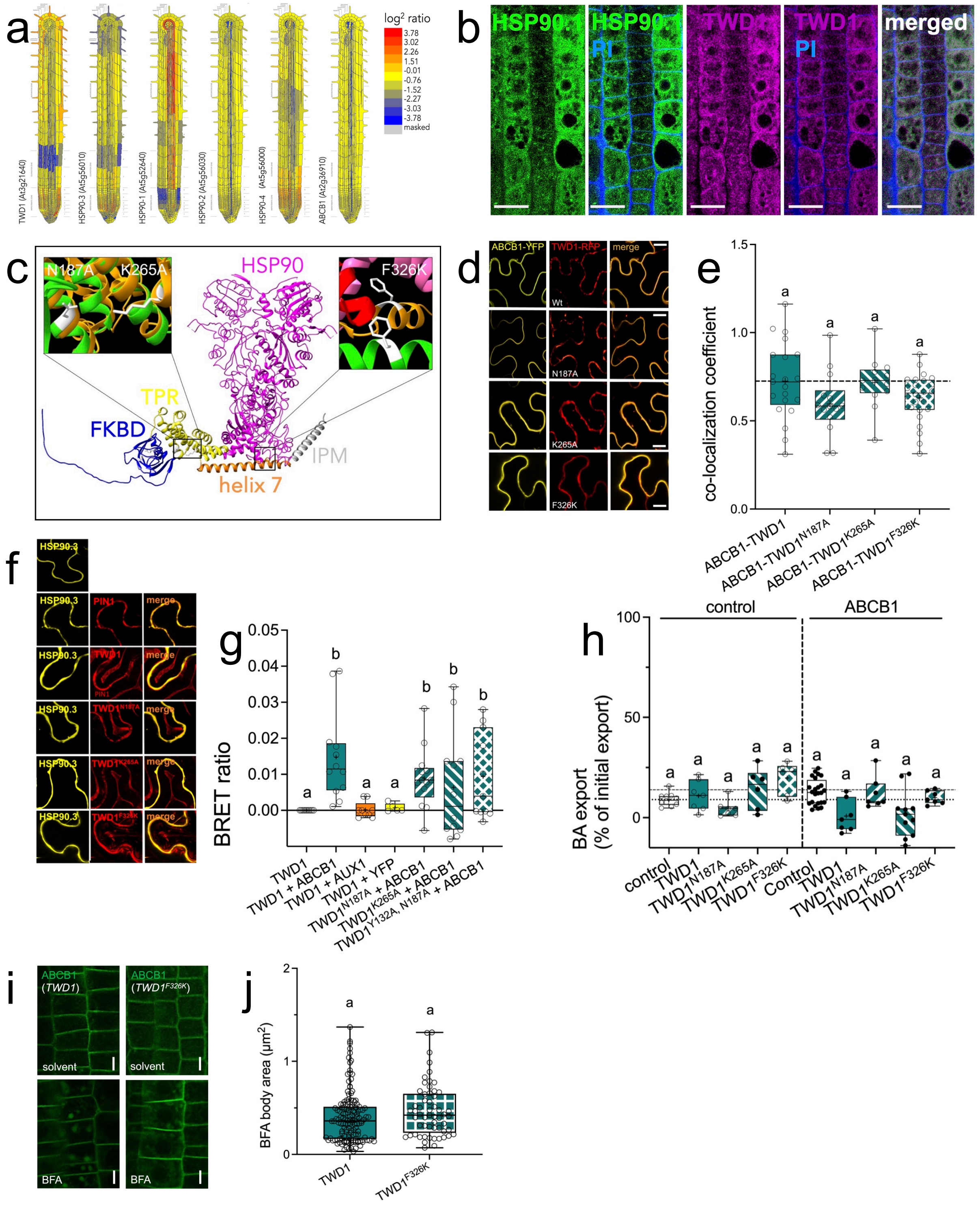

Supplementary Fig. 1 I FKBP42/TWD1 colocalizes and interacts with HSP90.

(a) *In silico* analyses of *HSP90*, *TWD1* and *ABCB1* expression in the root taken from eFP browser (https://bar.utoronto.ca/efp2/Arabidopsis/Arabidopsis_eFPBrowser2.html)

(b) Confocal **i**maging of HSP90.1 (*HSP90:HSP90.1-mNeonGreen* and TWD1 (TWD1:TWD1-CFP; bars 200 µm.

(c) Hypothetical model of TWD1-HSP90 interaction and location of used TPR (N187A, K265A) and helix 7 (F326K) mutations.

(d-e) Co-expression with TPR mutations of TWD1 does not alter ABCB1 expression in tobacco based on imaging (**d**) and quantification of co-localization (**e**). Significant differences ($p < 0.05$) of means ± SE (n = 3 independent tobacco transfections with each 6-8 cells) were determined using Ordinary One-way ANOVA (Tukey's multiple comparison test) and are indicated by different lowercase letters; bar = 50 µm.

(f) HSP90-YFP and TWD1-RFP co-localization controls upon co-expression in tobacco leaves used for of FRET-FLIM measurements; bars = 50 µm.

(g) TPR helix 7 mutation of TWD1 does not interfere with ABCB1-TWD1 interaction analyzed by BRET in tobacco. Significant differences ($p < 0.05$) of means ± SE (n = 3 independent tobacco transfections) were determined using Ordinary One-way ANOVA (Dunnett's multiple comparisons test) and are indicated by different lowercase letters.

(h) TPR and helix 7 mutations of TWD1 are functional in activation of ABCB1-mediated IAA export in tobacco (benzoic acid (BA; diffusion) control). Significant differences ($p < 0.05$) of means ± SE (n = 10-18 independent protoplast preparations) were determined using Ordinary One-way ANOVA (Tukey's multiple comparison test) and are indicated by different lowercase letters.

(i-j) Confocal imaging (**i**) of ABCB:ABCB1-GFP (in *twd1-3*) lines complemented with indicated TPR and helix 7 mutations of *35S:TWD1-mCherry* treated with 50 µM BFA. Quantification of BFA body areas (**j**). Significant differences ($p < 0.05$) of means ± SE (n = 20-30 cells) were determined using Unpaired *t* test (Welch's correction) and are indicated by different lowercase letters; bars, 50 µm.
Data are presented as box-and-whisker plots, where median and 25th and 75th percentiles are represented by the box itself and the middle line, respectively; means are indicated by a "+". Source data are provided as a Source Data file.

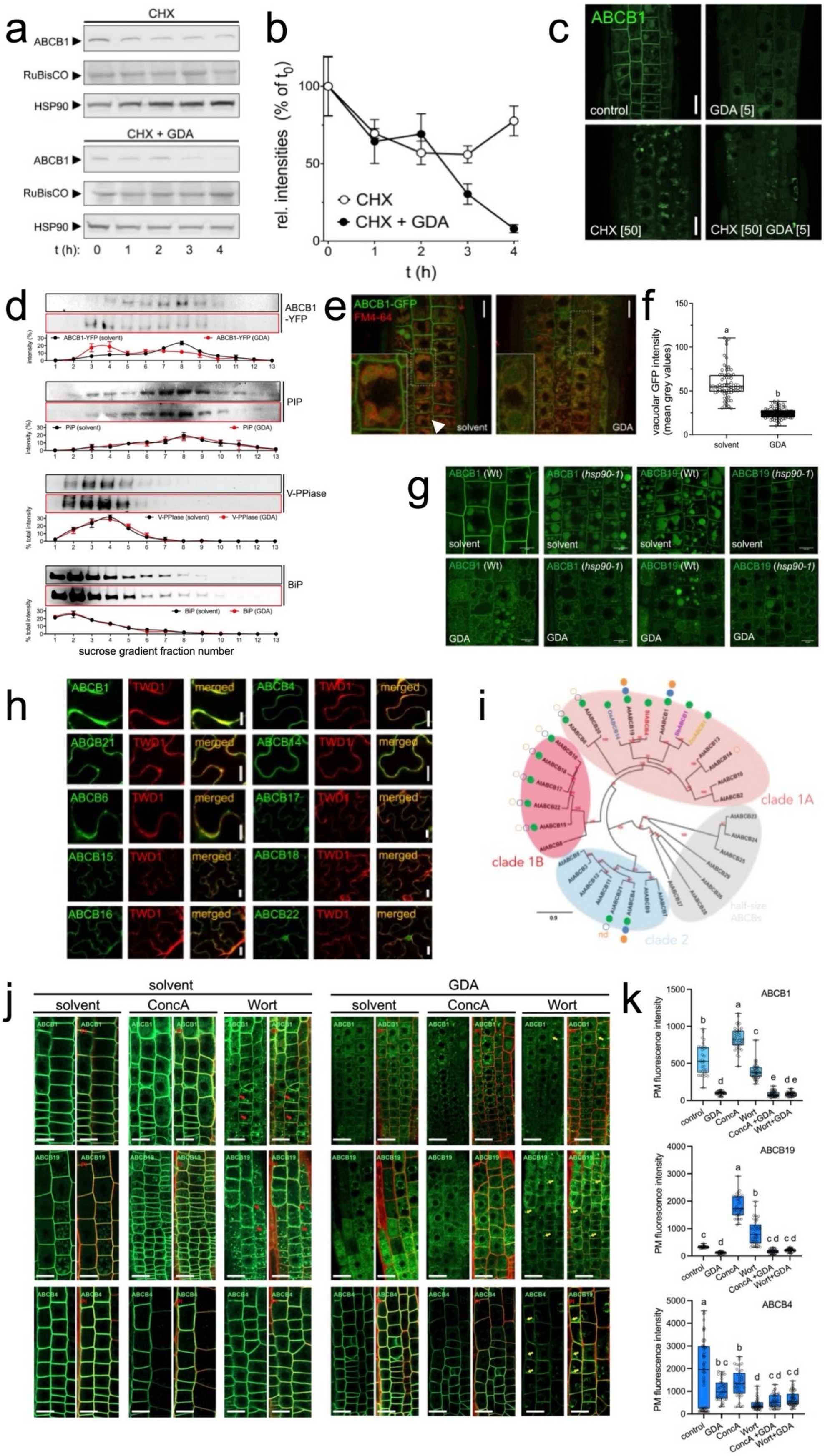

Supplementary Fig. 2 l Auxin-transporting ABCBs that interact with and that are regulated by TWD1 are HSP90 clients.

(a-b) Time-kinetics of ABCB1-GFP degradation by geldanamycin (GDA) in the presence and absence of cycloheximide (CHX, 50 µM). (**a**) Western (anti-GFP and anti-HSP90) and loading control (RuBiscCO Ponceau S stain) and (**b**) quantification of ABCB1 bands. n = 3 independent GDA treatments and Western analyses; an exemplary result is shown.

(c) ABCB1-GFP degradation by geldanamycin (GDA; 5 µM) in the presence and absence of cycloheximid (CHX; 50 µM); bar, 50 µm.

(d) Plasma membrane presence of ABCB1-GFP but not of plasma membrane marker, PIP, vacuolar marker, V-PPase, or ER marker, BiP, is sensitive to geldanamycin (GDA; 5 µM). Note that GDA treatment (red fractions) is accompanied by a shift from PM to vacuolar membranes revealed by Western analyses of fractions taken from a continuous 10-50% sucrose gradient.

(e-f) Plasma membrane destabilization of ABCB1-GFP by geldanamycin (GDA; 5 μM for 24h in the dark) treatment is accompanied by a shift from PM to vacuolar membranes marked by arrowheads, respectively; bar = 100 μm (**e**). Quantification of total vacuolar signals (**f**). Significant differences (p < 0.05) of means ± SE (n=3 independent GDA treatments with 75 vacuoles) were determined using Unpaired *t* test (Welch's correction) and are indicated by different lowercase letters.
(g) PM presence of ABCB1 and ABCB19 is reduced in *hsp90.1;* bars = 100 μm.
(h) ABCB-GFP and TWD1-mcherry colocalization control upon co-expression in tobacco; bars = 100 μm.
(i) Phylogenetic tree of Arabidopsis and selected plant ABCBs. Auxin-transport, TWD interaction and GDA sensitivity are indicated by filled green, blue and orange circles, respectively. Note that GDA sensitivity of ABCB21 could not be tested due to its low expression in roots.
(j) Confocal imaging of ABCB1, ABCB19 and ABCB4 in solvent and GDA-treated seedlings (GDA, 5 μM) after treatments with concanamycin (1 μM for 2h) and wortmannin (15 μM for 3h). Red and yellow arrows indicate mistargeting at the tonoplast and inhibition of endocytosis at the PM, respectively; bars = 100 μm.
(**k**) Quantification of PM fluorescence signals in the transition zone of the root tip, corresponding to PM-specific analysis of manually traced ROIs precisely outlining the plasma membrane to extract membrane-localized signals was performed using the Zen Blue 2012 software package. Significant differences (p < 0.05) of means ± SE (n=3 independent experiments with 75 cells) were determined using Unpaired *t* test (Welch's correction) and are indicated by different lowercase letters.
Data are presented as box-and-whisker plots, where median and 25th and 75th percentiles are represented by the box itself and the middle line, respectively; means are indicated by a "+". Source data are provided as a Source Data file.

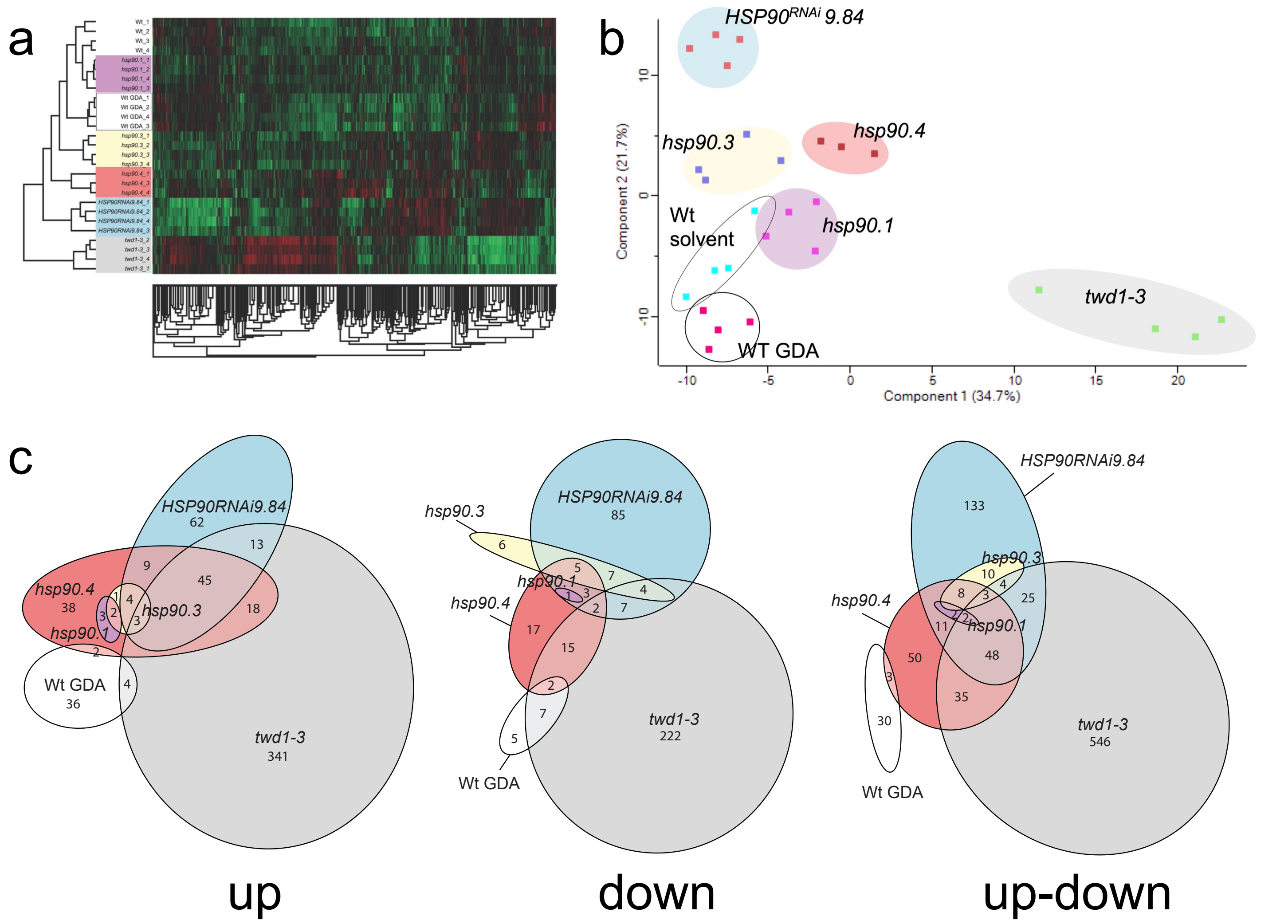

Supplementary Fig. 3 I Quality control and intersection analyses of TMT-based 16-channel multiplexing proteomics.

(a-b) Global analysis showing grouping of data based on genotypes and treatments. Heat map and hierarchical clustering; normalized abundances of each channel were log2 and z score transformed before analysis (**a**). Principal component analysis (**b**).

(c) Intersection (Euler) analyses of identified proteins that are significantly different (up-, down, or up and down-regulated) between the indicated 6 groups (pairwise *t*-test with an FDR of 0.05 and a fold change of at least 1.5).

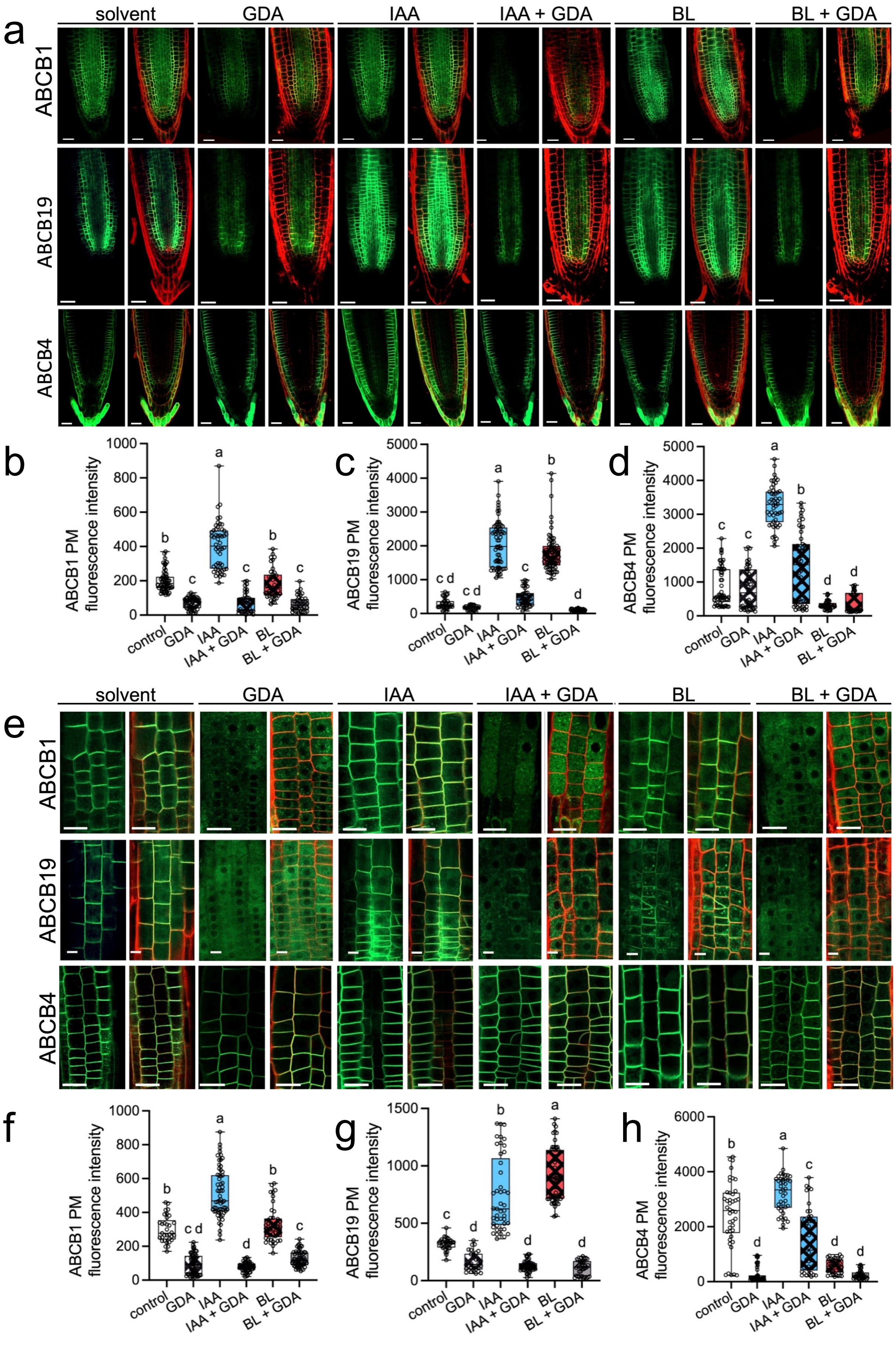

Supplementary Fig. 4 I IAA and BL differentially up-regulate the expression of ABCB1, ABCB19 and ABCB4. (**a-h**) PM presence of ABCB1, ABCB4, ABCB19 in the root tip (**a-d**) and root transition zone (**e-h**) upon solvent and GDA treatment of seedlings after the application of IAA (1µM for 2h) and BL (100 nM for 4h). Significant differences ($p < 0.05$) of means ± SE (n = 3 independent experiments with 40-50 cells) were determined using Ordinary One-way ANOVA (followed by Šídák's multiple comparisons test) and are indicated by different lowercase letters; bars = 100 µm. Quantification of PM fluorescence signals in the root tip (**b-d**) and transition zone of the root tip (**f-h**) corresponding to PM-specific analysis of manually traced ROIs precisely outlining the plasma membrane to extract membrane-localized signal was performed using the Zen Blue 2012 software package.

Data are presented as box-and-whisker plots, where median and 25th and 75th percentiles are represented by the box itself and the middle line, respectively; means are indicated by a "+". Source data are provided as a Source Data file.

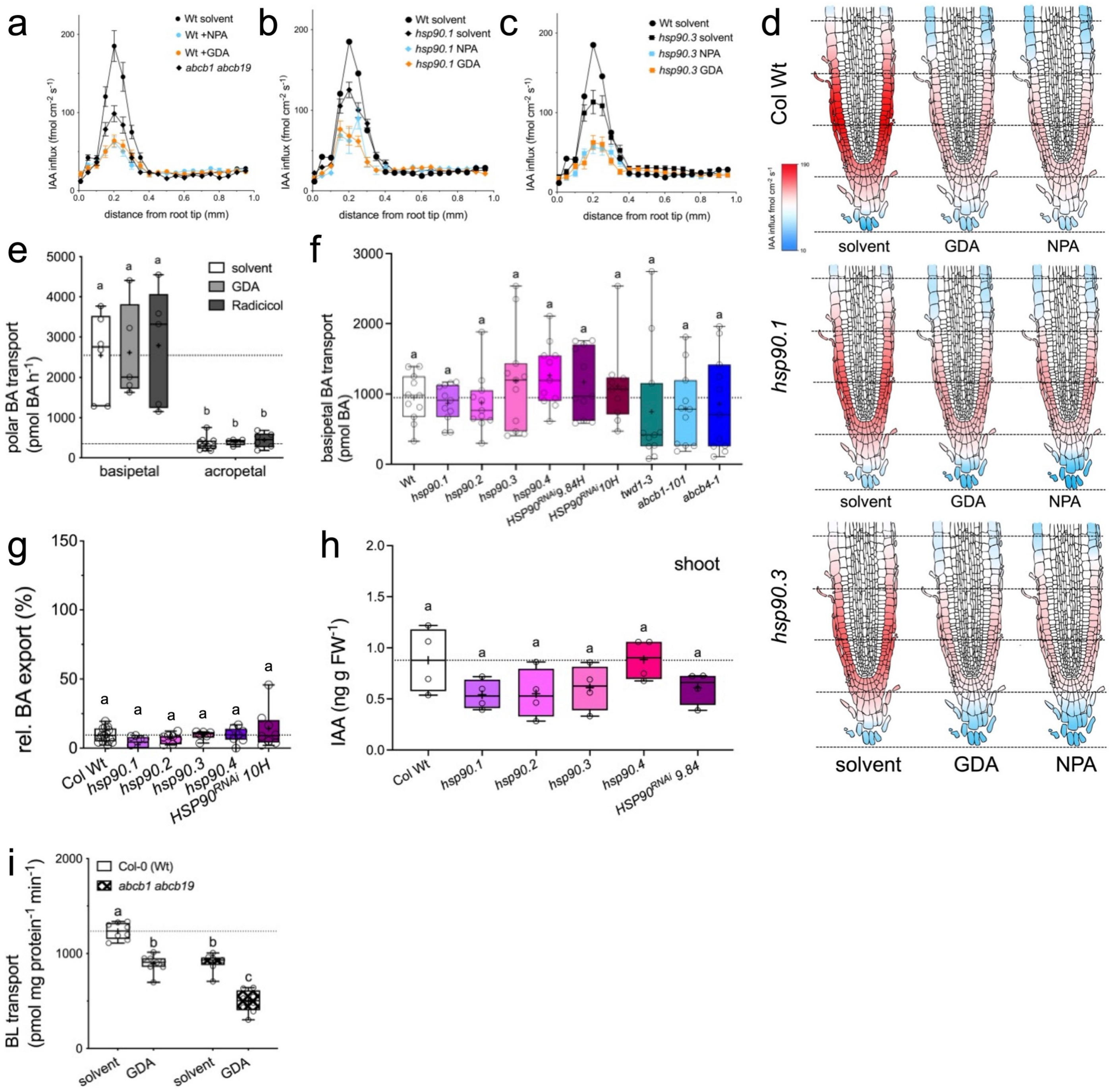

Supplementary Fig. 5 I HSP90 acts as positive regulators of root ABCB-mediated polar auxin transport

(a-d) Root IAA transport is likewise reduced by *ABCB* mutation as well as by NPA and Geldanamycin treatment (GDA; each 5 µM) measured by using an IAA-specific microelectrode. Influx profiles (**a-c**) and heat map presentation (**d**); dashed lines indicate 100-µm distances from the root tip.

(e) Benzoic acid (BA) diffusion control of root PAT in the presence and absence of GDA and Radicicol (each 5 µM). Significant differences ($p < 0.05$) of means ± SE (n = 6 independent experiments with each 10 seedlings) were determined using Ordinary One-way ANOVA (Tukey's multiple comparison test) and are indicated by different lowercase letters.

(f) Benzoic acid (BA) control of root shootward (basipetal) PAT of Fig. 3c. Significant differences ($p < 0.05$) of means ± SE (n = 3 independent experiments, with each with 5 seedlings) were determined using Ordinary One-way ANOVA (Dunnett's multiple comparisons test) and are indicated by different lowercase letters.

(g) Benzoic acid (BA) export from leaf mesophyll protoplasts. Significant differences ($p < 0.05$) of means ± SE (n = 6-10 independent protoplast preparations) were determined using Ordinary One-way ANOVA (Tukey's multiple comparison test) and are indicated by different lowercase letters; diffusion control of Fig. 3d.

(h) Auxin (IAA) levels quantified by GC-MS are not altered in *HSP90* mutant shoots. Significant differences ($p < 0.05$) of means ± SE (n = 4 independent experiments, with each with 10 seedlings) were determined using Ordinary One-way ANOVA (Dunnett's multiple comparisons test) and are indicated by different lowercase letters.

(i) BL import into microsomes prepared from root-enriched Wt or *abcb1 abcb19* material treated with geldanamycin (GDA) or solvent. Significant differences ($p < 0.05$) of means ± SE (n = 6 independent protoplast preparations) were determined using Ordinary One-way ANOVA (Tukey's multiple comparison test) and are indicated by different lowercase letters.

Data are presented as box-and-whisker plots, where median and 25th and 75th percentiles are represented by the box itself and the middle line, respectively; means are indicated by a "+". Source data are provided as a Source Data file.

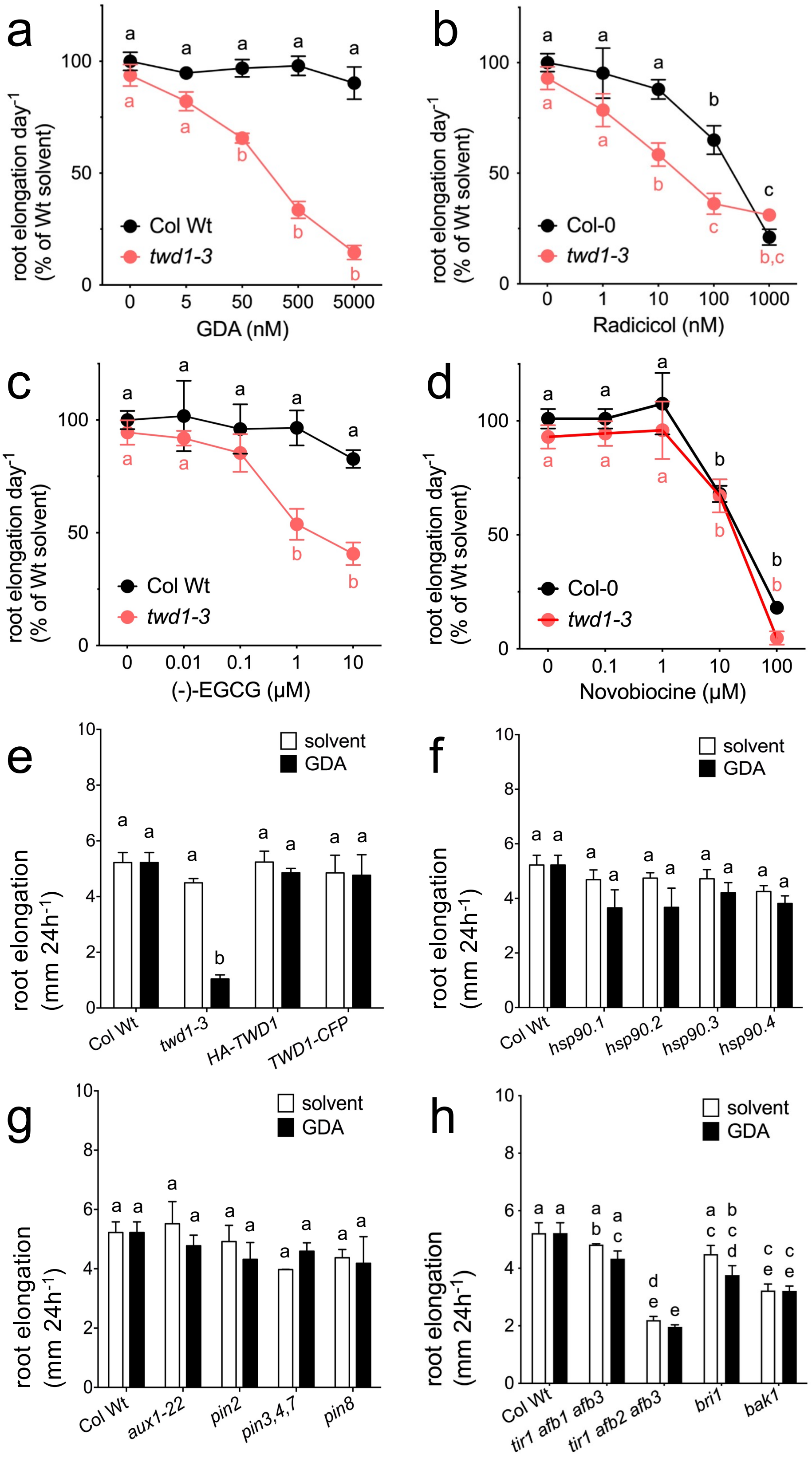

Supplementary Fig. 6 I HSP90 inhibition by geldanamycin affects *twd1* root elongation independent of transcriptional auxin responses and brassinosteroid signaling.

(a-d) Concentration-dependent effect of GDA (**a**), radicicol (**b**), (-)-epigallocatechin gallate ((-)-EGCG (**c**) and novobiocine (**d**) on relative root elongation of WT (Col W) and *twd1* (*twd1-3*) lines (each 5 µM; 24h).

(e) Effect of geldanamycin (GDA; 5 µM) on epidermal root elongation of *twd1* is complemented by 35S:*HA-TWD1* and *TWD1:TWD1-CFP.*

(f) Effect of geldanamycin (GDA; 5 µM) on root elongation of *hsp90* alleles is not significantly different to Wt; n=3.

(g) Effect of geldanamycin (GDA; 5 µM) on root elongation of *aux1* and *pin* mutant combinations is not significantly different to Wt.

(h) Effect of geldanamycin (GDA; 5 µM) on root elongation of a quadruple auxin receptor mutant and brassinosteroid mutants is not significantly different to Wt.

Significant differences (p < 0.05) of means ± SE (n = 3 independent experiments, with each 10 seedlings) were determined using Two-way ANOVA (Šídák's multiple comparisons test) and are indicated by different lowercase letters. Data are presented as box-and-whisker plots, where median and 25th and 75th percentiles are represented by the box itself and the middle line, respectively; means are indicated by a "+". Source data are provided as a Source Data file.

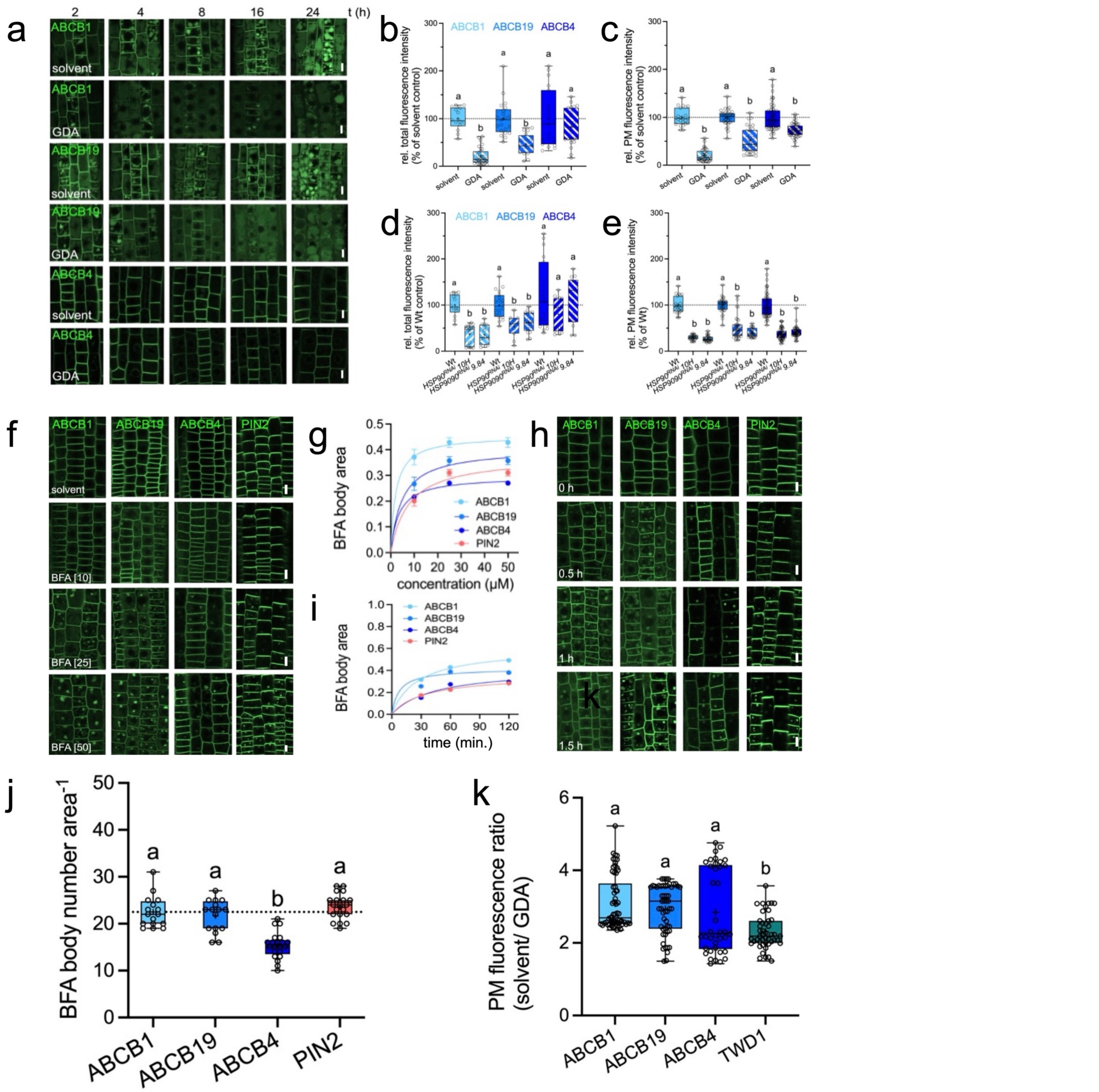

Supplementary Fig. 7 I Time and concentration course of Geldanamycin and BFA treatments of ABCBs.

(a) Time-course (0-24h) of geldanamycin (GDA; 5 µM) treatments of ABCB1-GFP, ABCB19-GFP and ABCB4-GFP in the transition zone of roots. Bar = 100 µm.

(b-c) Quantification of total fluorescence of cells of the root tip (**b**) and plasma membrane signals of cells of the transition zone (**c**) after Geldanamycin treatment (GDA; 5 µM). Significant differences ($p < 0.05$) of means ± SE (n = 3 independent GDA treatments with 14-20 cells) were determined using Ordinary One-way ANOVA (followed by Šídák's multiple comparisons test) and are indicated by different lowercase letters.

(d-e) *$HSP90^{RNAi}$* (lines *10H* and *9.84*) differentially destabilizes ABCB1, ABCB19 and ABCB4. Total GFP fluorescence of cells in the root tip (**d**) and plasma membrane signals of cells of the transition zone (**e**). Significant differences ($p < 0.05$) of means ± SE (n = 3 independent experiments with 14-20 cells) were determined using Ordinary One-way ANOVA (followed by Šídák's multiple comparisons test) and are indicated by different lowercase letters.

(f-g) Concentration-course of Brefeldin A (BFA) treatments of ABCB1, ABCB19 and ABCB4-GFP roots. Bar = 100 µm.

(h-i) Time-course of Brefeldin A (BFA, 25 µM) treatments of ABCB1, ABCB19 and ABCB4-GFP roots, bar = 100 µm.

(j) Quantification of BFA body numbers (50 µM) in the root tip. Data are from n=3 independent BFA treatments with the indicated number of imaged cells (**g, i, j**).

(k) Ratios of PM fluorescence of all three ABCBs and TWD1 in the absence and presence of GDA in the root transition zone. Significant differences ($p < 0.05$) of means ± SE (n = 20-30 cells) were determined using Ordinary One-way ANOVA (Tukey's multiple comparisons test) and are indicated by different lowercase letters.

Data are presented as box-and-whisker plots, where median and 25th and 75th percentiles are represented by the box itself and the middle line, respectively; means are indicated by a "+". Source data are provided as a Source Data file.

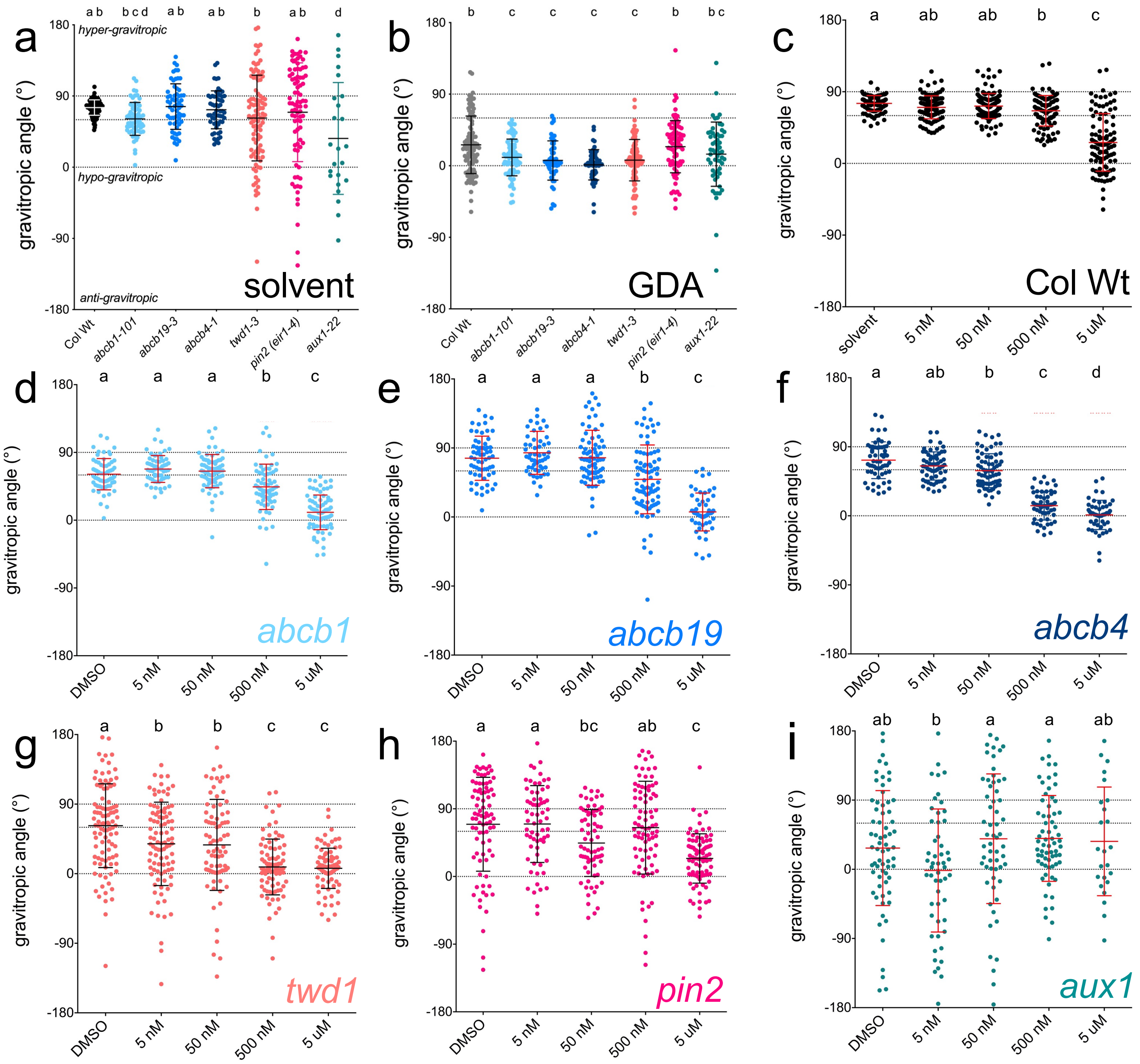

Supplementary Fig. 8 I Effect of HSP90 inhibition on gravitropic root bending

(a-i) Root bending of indicated mutant lines after re-orientation by 90°. GDA concentrations (24h treatment) in **a-b** were 5 µM, GDA concentrations in **c-i** are indicated. Significant differences of means (n=3 independent GDA treatments with each 20-30 seedlings) ± SD to solvent control were determined using Ordinary one-way ANOVA (Dunnett's multiple comparison) and are indicated by lowercase numbers.

Source data are provided as a Source Data file.

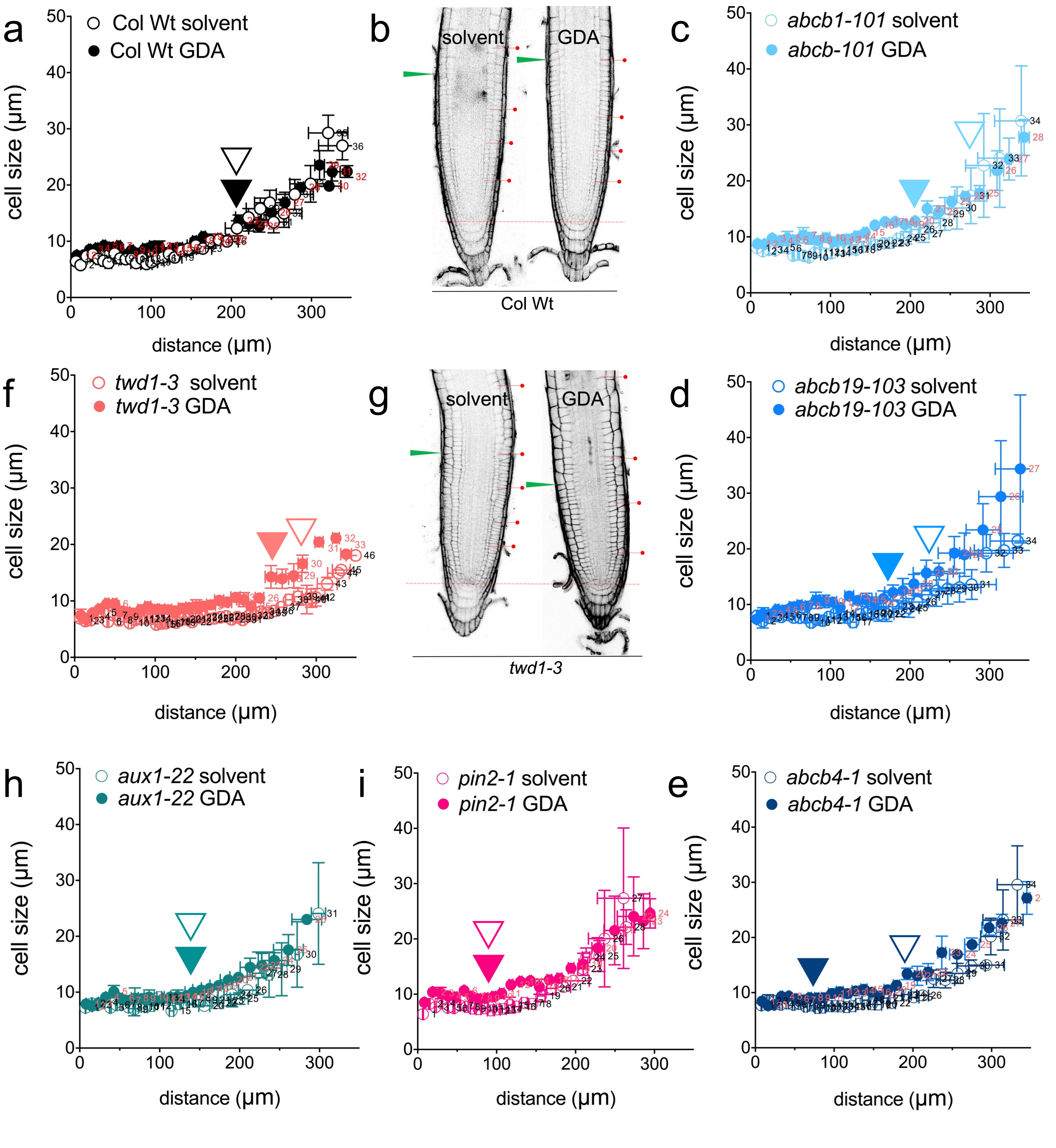

Supplementary Fig. 9 I Geldanamycin treatment shifts the onset of cell size increase root-ward in *abcb* and *twd1* mutant roots.

(a-e) Division zones of Wt (Col Wt) (**a**), *abcb1* (*abcb1-101*) (**c**), *abcb19* (*abcb19-103*) (**d**), *abcb4 (abcb4-1)* (**e**), *twd1* (*twd1-3*) (**f-g**), *pin2* (*eir1-4*) (**h**) and *aux1* (*aux1-22*) (**i**) roots grown on solvent control or geldanamycin (GDA; 5 µM) were propidium iodine stained and imaged by confocal microscopy. For reference, Wt (**b**) and *twd1* (**g**) confocal images are shown; onset of cell size increase is marked by green arrows. Cell sizes were determined from the root tip; n=3. Note the shift of the onset of cell elongation between solvent control (open triangles) and GDA treatments in *abcb* and *twd1* mutants (filled triangles). Shown are means ± SD from n=3 independent GDA treatments with each 20-30 seedlings).
