# Supplementary material for: HSP90 differentially stabilizes plant ABCB-type auxin transporters on the plasma membrane
Source: Nat Commun. 2025 Sep 30;16:8643. doi: 10.1038/s41467-025-63780-w (PMC12484997; doi:10.1038/s41467-025-63780-w)
Supplement: Supplementary file 2 — Description of Additional Supplementary Files [file 41467_2025_63780_MOESM2_ESM.docx]

Description of Additional Supplementary Files

**Supplementary Data 1:** Quality control of TMT-based 16-channel multiplexing proteomics data, related to Figure 2h.

Protein abundance of microsomal proteins quantified by TMT-based 16-channel multiplexing proteomics of Arabidopsis Wt (Col Wt) , *twd1-3*, *hsp90.1*, *hsp90.3*, *hsp90.4* and HSP90^RNAi^9.84 seedlings grown in liquid cultures. Wt was treated with either a solvent (WT solvent) or 5 μM geldanamycin (GDA) for 24h.
