# Supplementary material for: HSP90 differentially stabilizes plant ABCB-type auxin transporters on the plasma membrane
Source: Nat Commun. 2025 Sep 30;16:8643. doi: 10.1038/s41467-025-63780-w (PMC12484997; doi:10.1038/s41467-025-63780-w)
Supplement: Supplementary file 4 — Reporting Summary [file 41467_2025_63780_MOESM4_ESM.pdf]

nature portfolio

Corresponding author(s): Markus M. GEISLER

Last updated by author(s): 2025/06/20

Reporting Summary

Nature Portfolio wishes to improve the reproducibility of the work that we publish. This form provides structure for consistency and transparency in reporting. For further information on Nature Portfolio policies, see our Editorial Policies and the Editorial Policy Checklist.

Statistics

For all statistical analyses, confirm that the following items are present in the figure legend, table legend, main text, or Methods section.

n/a	Confirmed	
☐	☒	The exact sample size (*n*) for each experimental group/condition, given as a discrete number and unit of measurement
☐	☒	A statement on whether measurements were taken from distinct samples or whether the same sample was measured repeatedly
☐	☒	The statistical test(s) used AND whether they are one- or two-sided<br>*Only common tests should be described solely by name; describe more complex techniques in the Methods section.*
☒	☐	A description of all covariates tested
☐	☒	A description of any assumptions or corrections, such as tests of normality and adjustment for multiple comparisons
☐	☒	A full description of the statistical parameters including central tendency (e.g. means) or other basic estimates (e.g. regression coefficient) AND variation (e.g. standard deviation) or associated estimates of uncertainty (e.g. confidence intervals)
☐	☒	For null hypothesis testing, the test statistic (e.g. *F*, *t*, *r*) with confidence intervals, effect sizes, degrees of freedom and *P* value noted<br>*Give P values as exact values whenever suitable.*
☒	☐	For Bayesian analysis, information on the choice of priors and Markov chain Monte Carlo settings
☒	☐	For hierarchical and complex designs, identification of the appropriate level for tests and full reporting of outcomes
☒	☐	Estimates of effect sizes (e.g. Cohen's *d*, Pearson's *r*), indicating how they were calculated

Our web collection on statistics for biologists contains articles on many of the points above.

Software and code

Policy information about availability of computer code

Data collection	Images were acquired using laser scanning microscopes, dpm's as a basis for transport studies were acquired by scintillation counting, Western blots and petri dish plates were scanned using a commercially available scanner, pot-grown plants were imaged using a commercial digital camera, and plant roots were images using a light microscope as described in the Material an Methods section.
Data analysis	GraphPad Prism 10.4.2 for Mac was used for data analyses of transport, imaging and phenotypic studies.

For manuscripts utilizing custom algorithms or software that are central to the research but not yet described in published literature, software must be made available to editors and reviewers. We strongly encourage code deposition in a community repository (e.g. GitHub). See the Nature Portfolio guidelines for submitting code & software for further information.

Data

Policy information about availability of data

All manuscripts must include a data availability statement. This statement should provide the following information, where applicable:

- Accession codes, unique identifiers, or web links for publicly available datasets
- A description of any restrictions on data availability
- For clinical datasets or third party data, please ensure that the statement adheres to our policy

This article does not contain any original code. Requests for data should be made to and will be fulfilled by M.M. Geisler (markus.geisler@unifr.ch), provided the

data will be used within the scope of the originally provided informed consent. Source data are provided with this paper.

Research involving human participants, their data, or biological material

Policy information about studies with human participants or human data. See also policy information about sex, gender (identity/presentation), and sexual orientation and race, ethnicity and racism.

Reporting on sex and gender	n/a
Reporting on race, ethnicity, or other socially relevant groupings	n/a
Population characteristics	n/a
Recruitment	n/a
Ethics oversight	n/a

Note that full information on the approval of the study protocol must also be provided in the manuscript.

Field-specific reporting

Please select the one below that is the best fit for your research. If you are not sure, read the appropriate sections before making your selection.

[x] Life sciences [ ] Behavioural & social sciences [ ] Ecological, evolutionary & environmental sciences

For a reference copy of the document with all sections, see nature.com/documents/nr-reporting-summary-flat.pdf

Life sciences study design

All studies must disclose on these points even when the disclosure is negative.

Sample size	The sample size for all experiments (n) is indicated in the figure legends.
Data exclusions	No data exclusion was performed.
Replication	All experiments were conducted as multiple, independent replicates as indicated in the figure legends.
Randomization	Randomization is not relevant to our study.
Blinding	Blinding is not relevant to our study.

Reporting for specific materials, systems and methods

We require information from authors about some types of materials, experimental systems and methods used in many studies. Here, indicate whether each material, system or method listed is relevant to your study. If you are not sure if a list item applies to your research, read the appropriate section before selecting a response.

Materials & experimental systems

n/a	Involved in the study	
[ ]	[x]	Antibodies
[x]	[ ]	Eukaryotic cell lines
[x]	[ ]	Palaeontology and archaeology
[x]	[ ]	Animals and other organisms
[x]	[ ]	Clinical data
[x]	[ ]	Dual use research of concern
[ ]	[x]	Plants

Methods

n/a	Involved in the study	
[x]	[ ]	ChIP-seq
[x]	[ ]	Flow cytometry
[x]	[ ]	MRI-based neuroimaging

Antibodies

Antibodies used	Anti-RFP (Red Fluorescent Protein; mouse monoclonal; Agrisera Product no: AS15 3033) and anti-GFP (Rabbit polyclonal antibody to Green Fluorescent Protein, ChromoTek Cat No. pabg1) was used for Western blot analyses.

Validation

Reactivity of anti-RFP (https://www.agrisera.com/cgi-bin/ibutik/SkapaFaktura.pl?SkrivPDF=J&Sprak=EN&artnr=AS15%203033&Friendly=red-fluorescence-protein-tag-mouse-monoclonal&skrivpdf=j&artgrp=1030&Friendly_Grupp=&funk=visa_artikel) and anti-GFP (https://www.ptglab.com/products/pictures/pdf/Fluorescent-protein-specificity-table-Antibodies.PDF) for Arabidopsis thaliana was confirmed by the manufacturer.

Dual use research of concern

Policy information about [dual use research of concern](#)

Hazards

Could the accidental, deliberate or reckless misuse of agents or technologies generated in the work, or the application of information presented in the manuscript, pose a threat to:

No	Yes	
☒	☐	Public health
☒	☐	National security
☒	☐	Crops and/or livestock
☒	☐	Ecosystems
☒	☐	Any other significant area

Experiments of concern

Does the work involve any of these experiments of concern:

No	Yes	
☒	☐	Demonstrate how to render a vaccine ineffective
☒	☐	Confer resistance to therapeutically useful antibiotics or antiviral agents
☒	☐	Enhance the virulence of a pathogen or render a nonpathogen virulent
☒	☐	Increase transmissibility of a pathogen
☒	☐	Alter the host range of a pathogen
☒	☐	Enable evasion of diagnostic/detection modalities
☒	☐	Enable the weaponization of a biological agent or toxin
☒	☐	Any other potentially harmful combination of experiments and agents

Plants

Seed stocks

The following Arabidopsis thaliana lines in ecotype Wassilewskija (Wt) were used: twd1-1 (At3g21640; (Geisler et al. 2003)); TWD1:TWD1-CFP (Wu et al. 2007); ABCB1:ABCB1-GFP, ABCB19:ABCB19-GFP (Mravec et al. 2008) and hsp90.4/shephard. Pin2/eir1-4 (At5g57090; (Luschnig et al. 1998); twd1-3 (Geisler et al. 2003); abcb19-3 (Lewis et al. 2007); abcb1-100 abcb19-3 (Wu et al. 2007); ABCB4:ABCB4-GFP (At2g47000; (Cho et al. 2007); 35S:HA-TWD1 (Geisler et al. 2003); PIN2:PIN2-GFP (Abas et al. 2006); DII:VENUS (Brunoud et al. 2012); hsp90.1 (At5g52640; SALK_007614), hsp90.2 (At5g56030; SALK_038646); hsp90.3 (At5g56010; SALK_015240) and hsp90.4 (At5g56000; SALK_084059 (Ishiguro et al. 2002)) were all in the Columbia Wt (Col-0).

Novel plant genotypes

To generate the HSP90:HSP90.1-neonGFP cassette, genomic DNA fragments of full-length HSP90.1 (At5g52640) without stop codon, including the promoter region of 2kb were amplified from Col-0 genomic DNA and cloned at the BamHI/XbaI sites of the modified pCambia1301 vector to get a neonGreen fusion. The HSP90:HSP90.1-neonGFP construct was transformed into the Agrobacterium tumefaciens strain GV3101 and Col-0 Wt Arabidopsis plants were transformed using the floral dip method.

Authentication

Isogenic, homozygous lines for the transgene in the F3 generations were used for further analyses.
